# miR 1296-5p Inhibits the Migration and Invasion of Gastric Cancer Cells by Repressing ERBB2 Expression

**DOI:** 10.1371/journal.pone.0170298

**Published:** 2017-01-18

**Authors:** Xia Shan, Wei Wen, Danxia Zhu, Ting Yan, Wenfang Cheng, Zebo Huang, Lan Zhang, Huo Zhang, Tongshan Wang, Wei Zhu, Yichao Zhu, Jun Zhu

**Affiliations:** 1 Department of Respiration, The Affiliated Jiangning Hospital of Nanjing Medical University, Nanjing, PR China; 2 Department of Thoracic Surgery, First Affiliated Hospital of Nanjing Medical University, Nanjing, PR China; 3 Department of Oncology, The Third Affiliated Hospital of Soochow University, Changzhou, PR China; 4 Safety Assessment and Research Center for Drug, Pesticide and Veterinary Drug of Jiangsu Province, Nanjing Medical University, Nanjing, PR China; 5 Department of Gastroenterology, First Affiliated Hospital of Nanjing Medical University, Nanjing, PR China; 6 Department of Oncology, First Affiliated Hospital of Nanjing Medical University, Nanjing, PR China; 7 Department of Physiology, Nanjing Medical University, Nanjing, PR China; 8 State Key Laboratory of Reproductive Medicine, Nanjing Medical University, Nanjing, PR China; 9 Department of Radiation Oncology, Jiangsu Cancer Hospital, Nanjing, PR China; University of South Alabama Mitchell Cancer Institute, UNITED STATES

## Abstract

The metastasis of gastric cancer, one of the most common tumors, has a molecular mechanism that is still largely unclear. Here we investigated the role of possible tumor-suppressor miR-1296-5p in the cell migration and invasion of ERBB2-positive gastric cancer. It found that miR-1296-5p was significantly down-regulated in gastric cancer tissues. Moreover, it was down-regulated in lymph node metastatic gastric cancer tissues compared with non-metastatic gastric cancer tissues. The luciferase activity of ERBB2 3'-untranslated region-based reporters constructed in SNU-216 and NUGC-4 gastric cancer cells suggested that *ERBB2* was the target gene of miR-1296-5p. Overexpressed miR-1296-5p reduced its target protein level and Rac1 activation, and inhibited the migration and invasion of SNU-216 and NUGC-4 gastric cancer cells. MiR-1296-5p was down-regulated in ERBB2-positive gastric cancer tissues compared with ERBB2-negative gastric cancer tissues. In ERBB2-positive gastric cancers, the miR-1296-5p expression was suppressed in a majority of metastatic lymph node tissues compared to non-metastatic gastric cancer samples. The migration and invasion of gastric cancer cells was inhibited by miR-1296-5p overexpression or herceptin treatment, and rescued by the overexpression of constitutively active Rac1-Q61L or ERBB2. Taken together, our findings first suggest that miR-1296-5p might be involved in the regulation on the migration and invasion of human gastric cancer cells at least in part via targeting ERBB2/Rac1 signaling pathway.

## Introduction

Gastric cancer, regarded as the culprit for cancer-caused deaths, is particularly rampant across Asia [1]. Despite the fact that advances have been made over the last decade, the underlying molecular mechanism of gastric cancer metastasis remains obscure [1, 2]. Currently, the signaling pathway in its metastatic cascade has shifted into the spotlight of relevant studies that are organized to find a new treatment for gastric cancer and therefore lower its mortality [3].

MicroRNAs (miRNAs), a strain of 22-nt noncoding RNAs, play a decisive role in posttranscriptional gene regulation of gastric cancer, pertaining to cell survival, proliferation, differentiation, migration, invasion and metastasis [2]. For instance, miR-1296 has been proved of its potential linkage with breast cancer chemoresistance and self-renewal capability [4]. In triple negative breast cancer (TNBC) cell lines and tissues samples, miR-1296 expression was significantly suppressed and increased the sensitiveness of TNBC cells to cisplatin treatment [5]. MiR-1296 was found significantly downregulated in prostate cancer tissues, bringing about an obvious decrease in the S phase of the cell cycle [6]. Not only that, in the apoptosis triggered by PI (a novel synthesized small-molecule compound) in the condition of cervical cancer, miR-1296 could make changes in the PIM1-STAT3 pathway [7]. In spite of these findings, more efforts should be done to reveal how specific miRNAs alter the migration of gastric cancer cells.

ERBB2 (also known as HER2) is a receptor tyrosine kinase (RTK) of the ERBB RTK family [8, 9]. Other members of this family include the epidermal growth factor receptor (EGFR or HER), ERBB3 (HER3) and ERBB4 (HER4) [8, 9]. Enough studies have reported that ERBB2 signaling can raise the capability of cancer cells to migrate and colonize the metastasis loci [10–13]. However, the potential effect of ERBB2 and its downstream pathway engaged in the modulation of cell migration in gastric cancer has not yet been reported. Here, we suggest for the first time that the miR-1296-5p level is inversely correlated with ERBB2 expression in gastric cancers, and miR-1296-5p repressed ERBB2 expression in gastric cancer cells. We further advocate that miR-1296-5p can inhibit cell migration by repressing ERBB2 expression and Rac1 activation in gastric cancer cells. Collectively, these findings cement the fact that miR-1296-5p is a novel regulator of ERBB2 in gastric cancers.

## Materials and Methods

### Clinical samples

Tissue samples of 106 patients with gastric cancer (106 gastric cancer tissues and 30 adjacent normal tissues) from the First Affiliated Hospital of Nanjing Medical University from 2013 to 2016 were recruited in this study. All tumor tissues with higher tumor cell density were histopathologically confirmed by a pathologist for RNA isolation and immunohistochemistry (IHC). All the samples were pathologically examined and stored in liquid nitrogen for miRNA analysis. The stage and histological type of the tumor were defined according to the 7th edition of the American Joint Committee on Cancer tumor-node-metastasis (TNM) staging system [14]. Ethical approval of the study was granted by the Clinical Research Ethics Committee, Nanjing Medical University. Written informed consent was taken from each participant.

### Cell culture and transient transfections

The human ERBB2-positive gastric cancer cell lines, SNU-216 and NUGC-4, were purchased from the Cell Bank of Shanghai (Shanghai, China). Cells were routinely cultured in RPMI 1640, supplemented with 10% fetal bovine serum (Hyclone, Logan, UT), at 37°C in a humidified atmosphere with 5% CO_2_. The cells were seeded in 6-well plates (Costar, Corning, NY) and cultured to 80–90% confluence, and then transiently transfected with miRNAs or plasmids using Lipofectamine 2000 Reagent (Invitrogen, Carlsbad, CA) in serum-free OPTI-MEM according to the manufacturer’s instructions. The cells were switched to fresh medium containing 10% FBS 6 h after the transfection and cultured for 48 h.

### Quantitative real-time PCR analysis for miRNA

Gastric cancer tissues and cells were isolated with Trizol reagent (Invitrogen, Carlsbad, CA) and miRNA fraction was further purified using a mirVana^™^ miRNA isolation kit (Ambion, Austin, TX). The concentration and purity of the RNA samples were determined spectroscopically. Expression of mature miRNA was assayed using stem-loop RT followed by real-time PCR analysis. The SYBR and U6 gene were used for detecting the gene amplification and normalizing the each sample, respectively. The primers of reverse transcription and polymerase chain reaction were purchased from RiboBio Co., Ltd (Guangzhou, China) named Bulge-Loop ^™^ miRNA qRT-PCR Primer Set as previously described [15]. qRT-PCR was performed according to the protocol of the primer set. PCR product amplification was detected by the level of fluorescence emitted by SYBR Green (SYBR^®^ Premix Ex Taq™ II, TaKaRa) which intercalated into double stranded DNA [15]. The ΔCt method was used for miRNA expression analysis of biopsy specimens. First, the cycle number at the threshold level of fluorescence (Ct) for each sample was determined. Next, the ΔCt value was calculated. The ΔCt value was the difference between the Ct value of miR-1296-5p and the Ct value of U6: ΔCt = Ct (miR-1296-5p)—Ct (U6). The fold-change for miRNA from cells relative to each control cells was calculated using the 2^-ΔΔCt^ method. PCR was performed in triplicate.

### Cell migration and invasion assays

Cell migration and invasion was assessed in a modified Boyden chamber (Costar), in which the two chambers were separated by a polycarbonate membrane (pore diameter, 8.0 mm) [16]. For cell migration assays, Boyden chamber wells were coated with human collagen I (10 mg/mL) for 1 h at 37°C. For cell invasion assays, Boyden chamber wells were coated with matrigel (BD Biosciences) for 30 min at 37°C. SNU-216 and NUGC-4 cells transfected with indicated miRNAs or plasmids were grown to subconfluence in tissue culture plates, then detached and thereafter centrifuged and rendered into single cell suspensions in serum-free culture medium supplemented with 5 mg/mL BSA. The suspensions containing 5×10^4^ cells were added to wells with a membrane placed at the bottom. The cells were allowed to migrate and invade for 6 h at 37°C in this assay. Thereafter, the medium was discarded; stationary cells were removed with a cotton-tipped applicator and the membranes were cut out of the chamber and stained with 0.5% crystal violet. The response was evaluated in a light microscope by counting the number of cells that had migrated and invaded through the membrane.

### Small G-protein activation assay

For RhoA, Cdc42 and Rac1 activation assays, cells were seeded into 6-well plates and transfected with miR-1296-5p mimic or miRNA mimic control as described above. The experiments were then performed according to the manufacturer’s protocol (Cytoskeleton Inc., Denver, CO, USA). The activation of RhoA, Cdc42 or Rac1 was normalized to the miRNA mimic control group. RhoA, Cdc42 and Rac1 activation assays were performed in triplicate.

### Dual-luciferase activity assay

The 3'-UTR of human ERBB2 containing the putative target site for the miR-1296-5p was chemically synthesized and inserted at the *Xba*I site, immediately downstream of the luciferase gene in the pGL3-control vector (Promega, Madison, WI) by Integrated Biotech Solutions Co., Ltd (Shanghai, China), respectively. Twenty-four hours before transfection, cells were seeded into 24-well plates (1.5×10^5^ cells/well). 200 ng pGL3-ERBB2-3'-UTR plus 80 ng pRL-TK (Promega) were transfected in combination with 60 pmol of the miR-1296-5p mimic or miRNA mimic control, respectively. Luciferase activity was measured 24 hr after transfection using the Dual Luciferase Reporter Assay System (Promega). Firefly luciferase activity was normalized to renilla luciferase activity for each transfected well. Three independent experiments were performed in triplicate.

### Western blot analysis

Gastric cancer cells were planted into 6-well plates (6×10^5^ cells /well). 72 h after the transfection of miR-1296-5p mimic or miRNA mimic control, the cells were harvested and homogenized with lysis buffer. Total protein was separated by denaturing 10% SDS-polyacrylamide gel electrophoresis. Western blot analysis was performed as described [17]. The primary antibodies for ERBB2, Rac1 and GAPDH were purchased from Cell Signaling Technology (Danvers, MA). Protein levels were normalized to GAPDH.

### Statistical analysis

Each experiment was repeated at least 3 times. Numerical data were presented as mean±SD. The difference between means was analyzed with Student’s t-test. All statistical analyses were performed using SPSS 13.0 software (Chicago, IL). Differences were considered significant when *p* < 0.05.

## Results

### MiR-1296-5p is down-regulated in metastatic gastric cancer tissues

**Fig 1:**
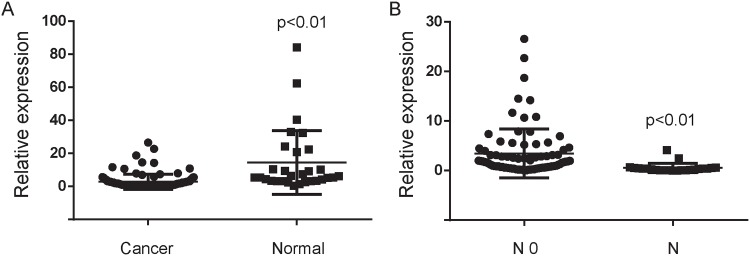
MiR-1296-5p is down-regulated in gastric cancer tissues. (A) The miR-1296-5p expression was suppressed in a majority of gastric cancer samples (n = 106) when compared to normal gastric samples (n = 30). The miRNA relative expression levels were normalized to the average value of gastric cancer samples. (B) The miR-1296-5p expression was suppressed in a majority of lymph node metastatic tissues (N, n = 23) when compared to non-metastatic gastric cancer samples (N0, n = 83). N0, no any lymph node metastases. N, lymph node metastases. The levels of miRNA relative expression were normalized to the average value of all gastric cancer samples.

With 106 cases of gastric cancer and 30 cases of non-tumor adjacent normal tissues, we used quantitative real-time PCR to justify whether miR-1296-5p expression was associated with gastric cancer. We found that miR-1296-5p expression was significantly down-regulated in gastric cancer tissues compared with the non-tumor adjacent normal tissues (Fig 1A). Meanwhile, we also found that miR-1296-5p was significantly down-regulated in lymph node metastatic tissues compared with non-metastatic gastric cancer tissues (Fig 1B). Therefore, miR-1296-5p was predicted as a tumor-suppressor in gastric cancer progress and metastasis.

### MiR-1296-5p suppresses the migration, invasion and Rac1 activation of gastric cancer cells

**Fig 2:**
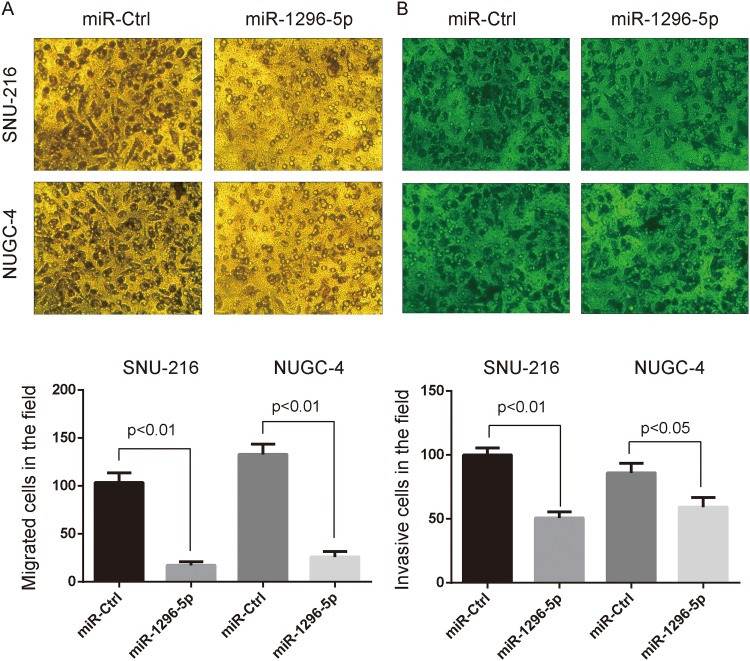
MiR-1296-5p suppresses the migration and invasion of gastric cancer cells. Human gastric cancer SNU-216 and NUGC-4 cells transiently transfected with miR-1296-5p or control miRNA were agree to migrate (A) and invade (B) in Boyden chambers. The top panel shows the gastric cancer cells migrated (A) and invaded (B) onto the bottom surface. Magnification, ×200. Statistic results are presented as mean±SD of 3 independent experiments.

In order to confirm the curbing role of miR-1296-5p in gastric cancer progress and metastasis, we transfected miR-1296-5p mimic into ERBB2-positive SNU-216 and NUGC-4 gastric cancer cells and then subjected them to cell migration and invasion assays. Less miR-1296-5p-overexpressing cells migrated and invaded onto the bottom surfaces of Boyden chambers significantly than the control miRNA-expressing cells (Fig 2).

**Fig 3:**
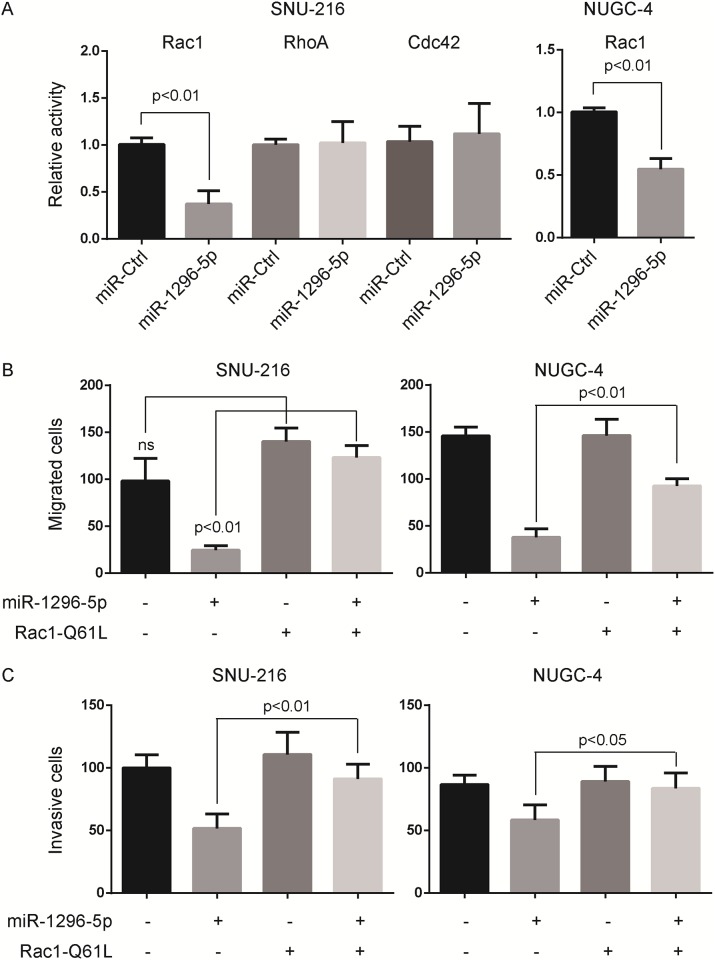
MiR-1296-5p suppresses Rac1 activation. (A) MiR-1296-5p overexpression inhibited the activation of Rac1, but not Cdc42 and RhoA, in SNU-216 and NUGC-4 gastric cancer cells. The relative active levels of RhoA, Rac1 or Cdc42 were normalized to the average value of SNU-216 or NUGC-4 cells transfected with control miRNA. (B and C) The migration and invasion of SNU-216 and NUGC-4 cells were inhibited by miR-1296-5p overexpression, and rescued by constitutively active Rac1-Q61L overexpression. The rates of cell migration (B) and invasion (C) were determined by Boyden chamber assays. ns, no significance.

Previous study reported that the migration and invasiveness of gastric cancer cells could not happen without the activation of the small GTPases [18]. Thus, we examined the activation of RhoA, Rac1 and Cdc42 in miR-1296-5p-transfected cells and the control cells. We found that it was the activation of Rac1, not RhoA and Cdc42, that was significantly inhibited in miR-1296-5p-transfected cells compared to that in control cells (Fig 3A). Moreover, the overexpression of miR-1296-5p did not alter the protein level of Rac1 (Supporting information file S1 Fig).

We next sought to determine whether Rac1 activation was needed in the cell migration and invasion of SNU-216 and NUGC-4 gastric cancer cells. Interestingly, we found that the migration of SNU-216 and NUGC-4 cells was inhibited by miR-1296-5p overexpression, and rescued by constitutively active Rac1-Q61L overexpression (Fig 3B). We also found that the invasion of SNU-216 and NUGC-4 cells was inhibited by miR-1296-5p overexpression, and rescued by constitutively active Rac1-Q61L overexpression (Fig 3C). Taken together, these experiments demonstrated that miR-1296-5p suppressed the Rac1 activation, the migration and invasion of gastric cancer cells.

### ERBB2 as a target gene of miR-1296-5p

**Fig 4:**
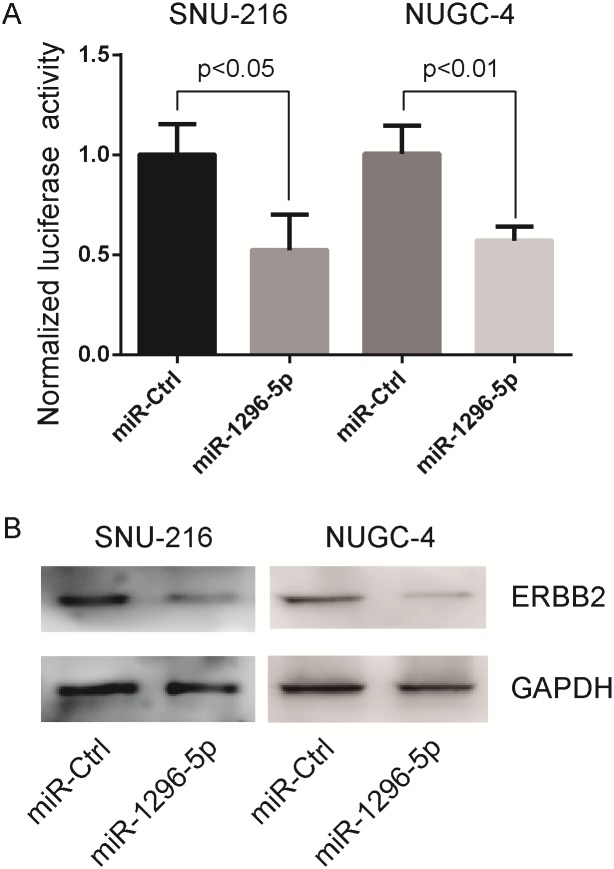
ERBB2 as a target of miR-1296-5p. (A) Luciferase assay showing reduction in reporter activity (relative luciferase units) after co-transfection of ERBB2-3’UTR with miR-1296-5p in SNU-216 and NUGC-4 cells. The luciferase activity was normalized to the average value of SNU-216 or NUGC-4 cells transfected with control miRNA. (B) Western blot analysis showing suppression of ERBB2 protein levels in SNU-216 and NUGC-4 cells after miR-1296-5p overexpression. GAPDH as loading control.

TargetScan Human (http://www.targetscan.org) predicted that *ERBB2* was the target gene of miR-1296-5p. To explore whether *ERBB2* was the target gene of miR-1296-5p, we constructed the luciferase reporter vectors with the putative *ERBB2* 3'-UTR target site for the miR-1296-5p downstream of the luciferase gene (pGL3-ERBB2-3'-UTR). Luciferase reporter vectors together with the miR-1296-5p mimic or the miRNA mimic control were transfected into SNU-216 and NUGC-4 cells, respectively. In gastric cancer cells, significant decrease of relative luciferase activity was noted when pGL3-ERBB2-3'-UTR was co-transfected with the miR-1296-5p mimic but not with the miRNA mimic control, respectively (Fig 4A). Next, we transfected the miR-1296-5p mimic and miRNA mimic control into SNU-216 and NUGC-4 cells and examined the expression of ERBB2. We found that the expression of ERBB2 in miR-1296-5p-transfected cells was extremely depressed than that in control cells (Fig 4B). Taken together, these results showed that *ERBB2* was the target gene of the miR-1296-5p.

### MiR-1296-5p is down-regulated in ERBB2-positive gastric cancer tissues

**Fig 5:**
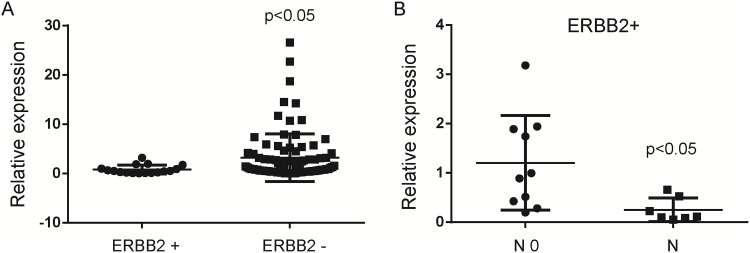
MiR-1296-5p is down-regulated in ERBB2-positive gastric cancer tissues. (A) The miR-1296-5p expression is suppressed in a majority of ERBB2-positive gastric cancer samples (n = 17) when compared to ERBB2-negative gastric cancer samples (n = 89). (B) In ERBB2-positive gastric cancers, the miR-1296-5p expression was suppressed in a majority of metastatic lymph node tissues (N, n = 7) when compared to non-metastatic gastric cancer samples (N0, n = 10). The miRNA relative expression levels were normalized to the average value of all gastric cancer samples. N0, no any lymph node metastases. N, lymph node metastases.

In view of the fact that *ERBB2* was the target gene of miR-1296-5p, we speculated that miR-1296-5p has negative correlation with ERBB2 in gastric cancer tissues. As expected, the miR-1296-5p expression was suppressed in a majority of ERBB2-positive gastric cancer samples compared to ERBB2-negative gastric cancer samples (Fig 5A). Interestingly, in ERBB2-positive gastric cancers, the miR-1296-5p expression was suppressed in a majority of metastatic lymph node tissues compared to non-metastatic gastric cancer samples (Fig 5B). These findings illustrated that miR-1296-5p was significantly down-regulated in ERBB2-positive gastric cancer tissues, especially in ERBB2-positive metastatic lymph node tissues.

### Constitutively active Rac1 rescues the ERBB2-mediated migration and invasion of gastric cancer cells

**Fig 6:**
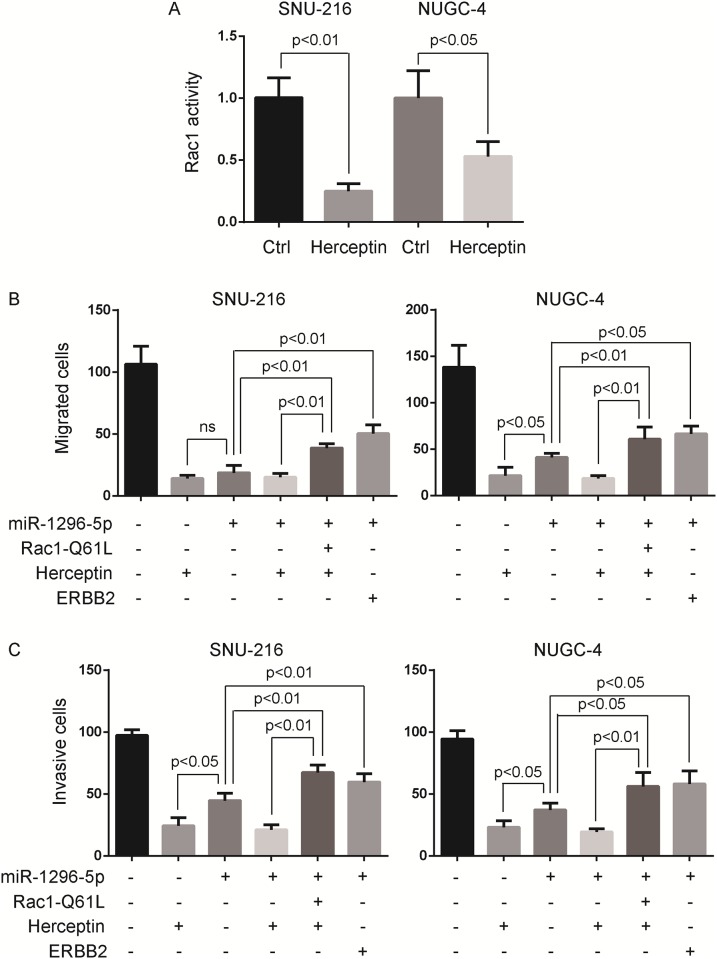
Constitutively active Rac1 rescues the ERBB2-mediated migration and invasion of gastric cancer cells. (A) Herceptin inhibited the Rac1 activation in SNU-216 and NUGC-4 gastric cancer cells. SNU-216 and NUGC-4 cells were treated with herceptin (1 nmol/L) for 24 h and then subjected to small G-protein activation assay. The relative active levels of Rac1 were normalized to the average value of SNU-216 or NUGC-4 cells treated with vehicle. (B) The migration of SNU-216 and NUGC-4 cells were inhibited by miR-1296-5p overexpression or herceptin treatment, and rescued by the overexpression of constitutively active Rac1-Q61L or ERBB2. The cell migration rate was determined by Boyden chamber assays. ns, no significance. (C) The invasion of SNU-216 and NUGC-4 cells were inhibited by miR-1296-5p overexpression or herceptin treatment, and rescued by the overexpression of constitutively active Rac1-Q61L or ERBB2. The cell invasion rate was determined by Boyden chamber assays.

We have found that miR-1296-5p suppressed the Rac1 activation (Fig 3A) and ERBB2 expression (Fig 4B). Afterwards, we determined whether the Rac1 activation was regulated by ERBB2 signaling. ERBB2-positivie gastric cancer cells, SNU-216 and NUGC-4 cells, were treated with herceptin (recombinant humanized anti-ERBB2 antibody) and then subjected to small G-protein activation assay. The Rac1 activation extremely declined after herceptin treatment (Fig 6A). Moreover, we examined the role of ERBB2 and Rac1 signaling on the migration and invasion of gastric cancer cells. Boyden chamber assays showed that the migration and invasion of SNU-216 and NUGC-4 cells were inhibited by miR-1296-5p overexpression or herceptin treatment, and rescued by the overexpression of constitutively active Rac1-Q61L or ERBB2 (Fig 6B and 6C). Herceptin treatment and miR-1296-5p overexpression had similar capability in retarding gastric cancer cell migration and invasion (Fig 6B and 6C). Together, these data strongly suggested that ERBB2 was inseparable to induce Rac1 activation and participated in the migration and invasion of gastric cancer cells.

## Discussion

Gastric cancer is one of the most frequently occurring malignancies in East Asian countries [1]. Metastasis remains the major cause of gastric cancer-related death. Elucidating the signaling pathways involved in the metastatic cascade has become a key goal for developing novel effective therapeutics aimed at reducing gastric cancer mortality rates. The first main observation in the present study is that miR-1296-5p expression is suppressed in a majority of gastric cancer samples when compared to that in normal gastric samples. MiRNA microarray analyses demonstrate that miR-1296 can affect PIM1-STAT3 pathway in PI003 (a novel synthesized small-molecule compound)-induced apoptosis in cervical cancer [7]. MiRNA-next generation sequencing analysis reveals the potential association of miR-1296 with breast cancer chemoresistance and self-renewal capability [4]. The other studies show that miR-1296 is down-regulated in prostate cancer and triple negative breast cancer (TNBC) samples [5, 6], suggesting that it might have a role as a tumor-suppressor gene in these cancers mentioned above. However, the role of miR-1296-5p as tumor-suppressor in cancer metastasis is still largely unknown.

This is the first study to demonstrate that miR-1296-5p is down-regulated in lymph node metastatic gastric cancer tissues. Moreover, miR-1296-5p is obviously down-regulated in ERBB2-positive gastric cancer samples compared to ERBB2-negative gastric cancer samples. In ERBB2-positive gastric cancers, the miR-1296-5p expression is more suppressed in a majority of metastatic lymph node tissues compared to non-metastatic gastric cancer samples, suggesting that miR-1296-5p might play a role of tumor metastasis suppressor in ERBB2-positive gastric cancer.

On cellular and molecular levels, miR-1296 overexpression plays a evidently suppressive role in the expression of Cyclin D1 and the multiplying of TNBC cell lines [5]. While in prostate cancer PC3 cells of human beings, the low expression of miR-1296 can upregulate both minichromosome maintenance 2 (MCM2) mRNA and protein, and conversely, its overexpression can lower the level of the both and even shorten the S phase of the cell cycle [6]. In this article, our experiment verifies the inhibitive role of miR-1296-5p in the migration and invasion of ERBB2-postive gastric cancer cells that is achieved by suppressing ERBB2 expression and Rac1 activation. In ERBB2-overexpressing cells, transforming growth factor beta (TGF-beta) recruits actin and actinin to HER2 that then activates Rac1 and promotes its motility and invasiveness [19].

Johnson et al. reported that ERBB2-induced migration cannot occur without the activation of the small GTPases Rac1 [20]. According to other studies, P130Cas/PI3K/Akt/Rac1 signaling pathway can function in ERBB2-dependent invasion in three-dimensional cultures of mammary epithelial cells [21]. Consistent with the results mentioned above, our data shows that ERBB2/Rac1 signaling regulates the migration and invasion of gastric cancer cells.

## Conclusions

In conclusion, the present study has partially clarified the association between miR-1296-5p and ERBB2-positive gastric cancer. To the best of our knowledge, this is the first study to demonstrate that miR-1296-5p might be involved in regulating the migration and invasion of human gastric cancer cells at least in part via targeting ERBB2/Rac1 signaling pathway. Therefore, miR-1296-5p is a candidate tumor suppressor miRNA molecule in gastric cancer and may be a potential clinical classification marker and therapeutic target for human gastric cancer metastasis.

## Supporting Information

S1 Fig MiR-1296-5p does not alter Rac1 protein level. Western blot analysis showing the unchanged of Rac1 protein levels in SNU-216 and NUGC-4 cells after miR-1296-5p overexpression. GAPDH as loading control.
(TIF)
