# Supplementary figures and images for: miR 1296-5p Inhibits the Migration and Invasion of Gastric Cancer Cells by Repressing ERBB2 Expression
Source: PLoS One. 2017 Jan 18;12(1):e0170298. doi: 10.1371/journal.pone.0170298 (PMC5242522; doi:10.1371/journal.pone.0170298)

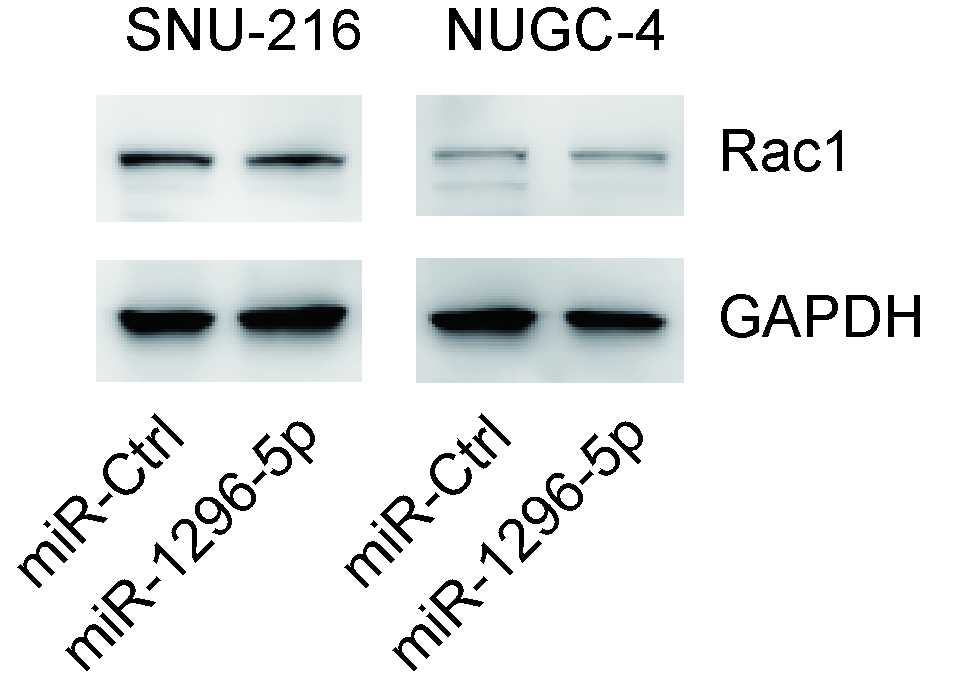

Supplement: S1 Fig — Western blot analysis showing the unchanged of Rac1 protein levels in SNU-216 and NUGC-4 cells after miR-1296-5p overexpression. GAPDH as loading control. (TIF) [file pone.0170298.s001.tif]
